# A modified survival model for patients with esophageal squamous cell carcinoma based on lymph nodes: A study based on SEER database and external validation

**DOI:** 10.3389/fsurg.2022.989408

**Published:** 2022-09-07

**Authors:** Tianbao Yang, Shijie Huang, Boyang Chen, Yahua Chen, Wei Liang

**Affiliations:** ^1^ Department of Cardiothoracic Surgery, The Affiliated Hospital of Putian University, Putian, China; ^2^ Department of Gastroenterology, The Affiliated Hospital of Putian University, Putian, China; ^3^ Department of Gastrointestinal Endoscopy, Fujian Provincial Hospital, Teaching Hospital of Fujian Medical University, Fuzhou, China

**Keywords:** nomogram, esophageal squamous cell carcinoma, examined lymph nodes, prognosis, decision curve analysis

## Abstract

**Background:**

The counts of examined lymph nodes (ELNs) in predicting the prognosis of patients with esophageal squamous cell carcinoma (ESCC) is a controversial issue. We conducted a retrospective study to develop an ELNs-based model to individualize ESCC prognosis.

**Methods:**

Patients with ESCC from the SEER database and our center were strictly screened. The optimal threshold value was determine by the X-tile software. A prognostic model for ESCC patients was developed and validated with R. The model’s efficacy was evaluated by C-index, ROC curve, and decision curve analysis (DCA).

**Results:**

3,629 cases and 286 cases were screened from the SEER database and our center, respectively. The optimal cut-off value of ELNs was 10. Based on this, we constructed a model with a favorable C-index (training group: 0.708; external group 1: 0.687; external group 2: 0.652). The model performance evaluated with ROC curve is still reliable among the groups. 1-year AUC for nomogram in three groups (i.e., 0.753, 0.761, and 0.686) were superior to that of the TNM stage (*P* < 0.05). Similarly, the 3-year AUC and the 5-year AUC results for the model were also higher than that of the 8th TNM stage. By contrast, DCA showed the benefit of this model was better in the same follow-up period.

**Conclusion:**

More than 10 ELNs are helpful to evaluate the survival of ESCC patients. Based on this, an improved model for predicting the prognosis of ESCC patients was proposed.

## Introduction

Esophageal squamous cell carcinoma (ESCC) is the most common histological form of esophageal cancer, which has made a major contribution to cancer-related mortality worldwide (1, 2). Remarkably, ESCC is mainly characterized by lymph node metastasis (LNM). Less than one-third of ESCC patients are able to cross the 5-year survival period (3–5). Due to the complex lymphatic network in and around the esophagus, the possible LNM of ESCC involves multiple fields, including the neck, chest, or/and abdomen (6–8). Therefore, radical lymphadenectomy for ESCC is regarded as an important method to improve the survival rate.

The lymph node resection during cancer surgery is generally performed for 2 main reasons, (a) staging and (b) dissemination prevention. Thus, the counts of resected nodes increases with the counts of suspicious nodes (up to a certain limit) and with the striving for dissemination prevention. In the first case, more nodes might indicate a bad prognosis, while in the latter, better dissemination prevention might be achieved by exciding more nodes. However, the counts of examined lymph nodes (ELNs) in predicting prognosis remains controversial (9–11). In addition, the American Joint Committee on Cancer indicated the number of ELNs was beneficial as many as possible (12–16).

Although ELNs were an independent factor for survival, there remained to be no associated study that reported the precision of the survival model for patients with ESCC based on the optimal threshold of ELNs. Factors such as age, grade, and tumor size may also significantly affect the prognosis of ESCC patients. Regarding these divergences and lack of relevant research, this study aimed to identify the optimal number of ELNs and build a nomogram model based on the grouping of ELNs by SEER database and data from our hospital. The optimal threshold value of ELNs was made out by X-tile software which was extensively used and credible for figuring out optimal cut-off values (17, 18). Through the SEER database and data collected from our hospital, we built and validated a nomogram model according to the results of multivariate cox analysis to predict the survival of ESCC patients. Combined with Cox analysis results of the data collected from SEER database and our hospital, a prediction model for patients with ESCC based on lymph nodes was established and verified.

## Material and methods

### Research material

The SEER database and the cases from our hospital were used to enroll patients. The SEER database the information was collected by SEER*Stat software (version 8.3.6), tumors with codes 8,070, 8,071, 8,072, 8,073, 8,074, 8,075, 8,076, and 8,078 were set as ESCC according to the ICD-O-3 criteria (19, 20).

We made the inclusion criteria for the SEER database: (1) patients aged over 20 years old and diagnosed as ESCC by histology; (2) patients who had detailed records of living status; (3) patients with valid information such as race, grading of tumors, ELNs, pathologic findings, and tumor size; (4) chemotherapy free before surgery. The following cases were excluded: the required information is missing or incomplete.

Cases were also selected from our center. Patients diagnosed from January 2016 to December 2019 were selected to analysis their information of diagnosis and treatment for ESCC. The criteria for including patients were: (1) Patients over 20 years of age with ESCC; (2) without preoperative adjuvant therapy. The exclusion criteria were: (1) no information on tumor progression or stage was available; (2) with chronic disease or organ dysfunction. Patients who did not participate in the follow-up were excluded. Tables 1, 2 show the data feature of SEER database and our center adoptive in this study respectively.

**Table 1:** Patients’ demographics, clinical characteristics at diagnosis.

Variables	Total (%)	2004–2009	2010–2015	*P* value
*n*	3,629	1,732	1,897	
**Age**				
<60	1,077 (29.68%)	516 (29.79%)	561 (29.57%)	0.415
≥60	2,552 (70.32%)	1,216 (70.21%)	1,336 (70.43%)	
**Race**				
White	731 (67.13%)	1,085 (62.64%)	1,192 (62.83%)	0.993
Black	220 (20.2%)	441 (25.46%)	481 (25.36%)	
Other	138 (12.67%)	206 (11.9%)	224 (11.8%)	
**Sex**				
Male	2,349 (64.73%)	1,130 (64.57%)	1,219 (64.26%)	0.536
Female	1,280 (35.27%)	602 (35.43%)	678 (35.74%)	
**Pathology grade**				
Well	207 (5.7%)	83 (4.79%)	124 (6.54%)	0.052
Moderately	1,875 (51.67%)	880 (50.81%)	995 (52.45%)	
Poorly	1,517 (41.8%)	754 (43.53%)	763 (40.22%)	
Undifferentiated	30 (0.83%)	15 (0.87%)	15 (0.79%)	
**Lymph node metastasis**				
No	1,654 (45.58%)	848 (48.96%)	806 (42.49%)	0.000
Yes	1,975 (54.42%)	884 (51.04%)	1,091 (57.51%)	
**Metastasis**				
No	2,748 (75.72%)	1,309 (75.58%)	1,439 (75.86%)	0.845
Yes	881 (24.28%)	423 (24.42%)	458 (24.14%)	
**Tumor size**				
≤3 cm	895 (24.66%)	424 (24.48%)	471 (24.83%)	0.172
>3 cm	2,734 (75.34%)	1,308 (75.52%)	1,426 (75.17%)	
**Examined LNs**				
≤10	3,052 (84.1%)	1,441 (83.2%)	1,611 (84.92%)	0.156
>10	577 (15.9%)	291 (16.8%)	286 (15.08%)	
**T stage**				
T1	422 (11.63%)	187 (5.15%)	235 (12.39%)	0.00
T2	1,428 (39.35%)	623 (35.97%)	805 (43.49%)	
T3	1,024 (28.21%)	526 (30.37%)	498 (26.25%)	
T4	755 (20.8%)	396 (22.86%)	359 (18.92%)	
**8th TNM stage**				
I	520 (14.33%)	276 (13.91%)	244 (12.86%)	0.051
II	1,033 (28.47%)	486 (41.89%)	547 (28.83%)	
III	1,200 (33.07%)	552 (35.43%)	648 (34.16%)	
IV	881 (24.28%)	423 (8.77%)	458 (24.14%)	
**Median survival (M)**	9 (4–23)	9 (4–23)	9 (4–22)	

**Table 2:** Patients’ demographics, clinical characteristics at diagnosis in our centre.

Variables	Patients from the our centre
*n*	268 (100%)
**Age**	
<60	89 (33.21%)
≥60	179 (66.79%)
**Sex**	
Male	222 (82.84%)
Female	46 (17.16%)
**Pathology grade**	
Well	17 (6.34%)
Moderately	16 (5.97%)
Poorly	44 (16.42%)
Unknown	191 (71.27%)
**Lymph node metastasis**	
No	160 (59.7%)
Yes	108 (42.3%)
**Metastasis**	
No	259 (96.64%)
Yes	9 (3.36%)
**Tumor size**	
≤3 cm	117 (43.66%)
>3 cm	151 (56.34%)
**Examined LNs**	
≤10	92 (34.33%)
>10	176 (65.67%)
**T stage**	
T1	36 (13.43%)
T2	32 (11.94%)
T3	155 (57.84%)
T4	45 (16.79%)
**8th TNM stage**	
I	36 (13.43%)
II	32 (11.94%)
III	191 (71.27%)
IV	9 (3.36%)
**Chemotherapy**	
No	244 (91.04%)
Yes	24 (8.96%)
**Smoking**	
No	95 (35.45%)
Yes	173 (64.55%)
**Drinking**	
No	123 (45.90%)
Yes	145 (54.10%)
**Median survival (M)**	28.5 (9–43)

### Variable definition

The clinicopathological variables included demographics, pathology, clinical stage, treatment, ELNs, and 8th TNM stage. In the latest version, some of the data were marked the status of TNM stage according to the 8th AJCC TNM system, while some data remained to be old edition. Therefore, after we abstracted the data, we transformed the old TNM staging system into the 8th AJCC TNM system because the number of positive examined lymph nodes and T stage were provided in the original data. Gender includes male and female. Age was converted to a dichotomous variable: <60 years and ≥60 years. Race mainly includes white, black and other races. The pathology was graded according to the degree of differentiation. LNM was recorded as positive (Y) and negative (N). Also, M1 indicated distant metastasis. The tumors were grouped according to their size as follows: ≤3 cm and >3 cm. While for ELNs, based on the result of X-tile software, the cut-off value was 10(18). Hence, ELNs were categorized into two groups: ≤10 and >10. Chemotherapy was described as Yes or No, as well as smoking. Overall survival (OS) and cancer-specific survival (CSS) were the main indicators.

### Statistical analysis

For data from the SEER database was investigated by the association among the categorical variables utilizing Pearson’s Chi-square test. a K-M survival curve was applied to analyze the OS and CSS according to the previous study (21). In addition, Univariate and multivariate Cox regression were used to determine the prognostic risk factors. After that, we build a nomogram model according to the results and validated it internally and externally. The cases from 2004 to 2009 were used as the training group, while the cases from 2010 to 2015 and the cases from our hospital were used as the validation group. C-index value, ROC curves, and decision curve analysis (DCA) were choosed to identify the value of model (22–24). All statistical analyses were performed using R version 4.1.3 and related packages. The difference was considered statistically significant when *P*-value <0.05.

## Results

### Basic characteristics

According to the flow chart (Supplementary Figures S1, S2), 3,629 patients diagnosed as ESCC from the SEER database were enrolled. We determined the diagnosis of ESCC based on pathological diagnosis, and then excluded patients with no information about TNM stage and survival status. As shown in Table 1, we included 3,629 patients from the SEER database including 1,732 patients from 2004 to 2009 and 1,897 patients from 2010 through 2015. According to Pearson’s Chi-square analysis, we found patients aged more than 50 years old accounted for a larger ratio than younger patients in ESCC patients, and male patients were more than female patients (*P* < 0.05). Furthermore, the total LNM rate was 54.42% and the distant metastasis rate was 24.28%. Accordingly, the median survival time was 9 months (range: ranged from 4 to 23 months). Also, we included 268 patients from our centre. The median survival time was 28.9 months (range: ranged from 9 to 43 months). In line with the SEER database, we also found patients with ESCC were inclined to be older people (66.79% vs. 33.21%) and male gender (82.84% vs. 17.16%). However, we found the rate of LNM and metastasis in our patients was lower than that in patients from the SEER database, which could be because our patients were diagnosed from 2016 to 2019 when endoscopy was extensively used.

### Grouping of ELNs in ESCC patients

**Figure 1:**
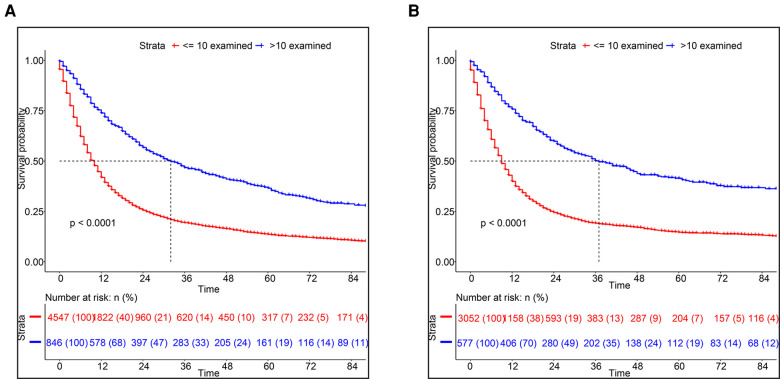
Kaplan-Meier survival analysis based on the number of ELNs. (**A**) OS, (**B**) CSS.

**Figure 2:**
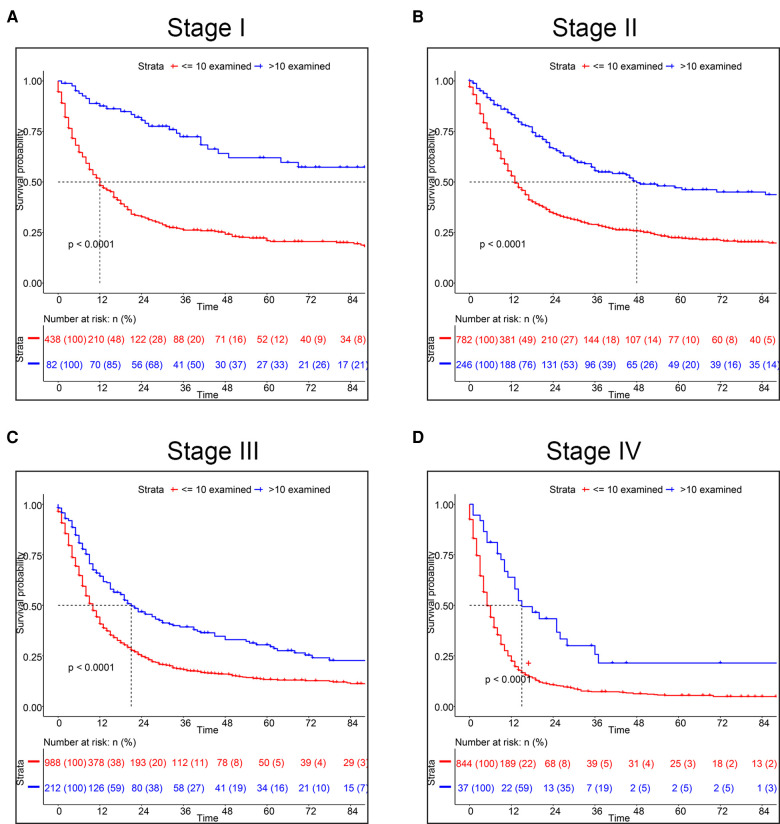
Kaplan-Meier survival analysis for CSS based on the number of ELNs. (**A**) Stage I, (**B**) Stage II, (**C**) Stage III, (**D**) Stage IV.

**Figure 3:**
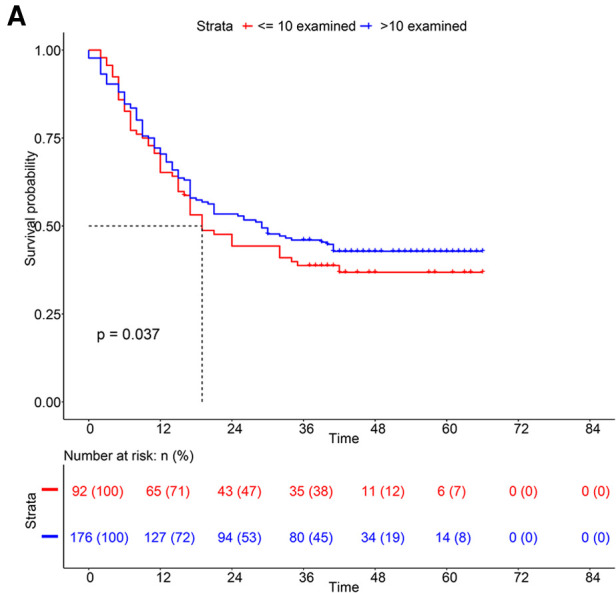
Kaplan-Meier survival analysis of patients from our center based on the number of ELNs.

Using X-tile software, we found the optimal cut-off of ELNs was 10 and divided into two groups (<10 vs. ≥10) (Supplementary Figure S3). As shown in Figure 1A, the OS rate between the two groups could be considered significantly different. Consequently, the CSS of patients with less than 10 ELNs was worse than patients with more than 10 ELNs (Figure 1B). Additionally, K–M survival analysis showed patients with >10 examined LNs who were in the different clinical TNM stages had better survival, of which the difference was statistically significant according to the grouping of ELNs (*P* < 0.0001) (Figure 2). Furthermore, to verify previous results, we analyzed whether the grouping of examined LNs was suitable for our clinical data. As shown in Figure 3, we found that patients with >10 examined LNs in our center had a higher survival rate (*P* = 0.037).

### Prediction model of ESCC survival

**Figure 4:**
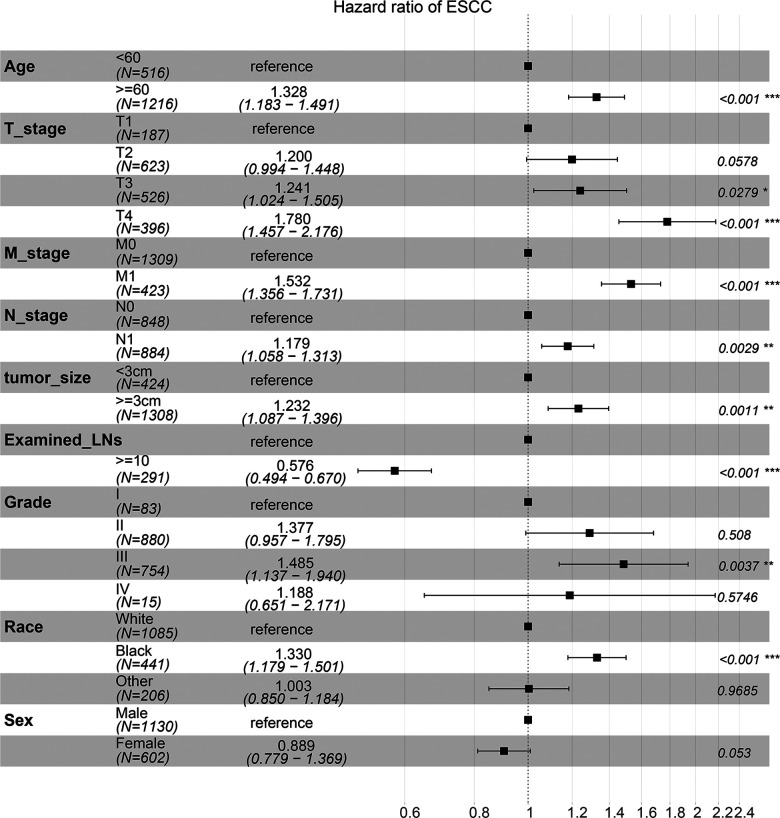
Multivariate Cox regression analysis.

**Figure 5:**
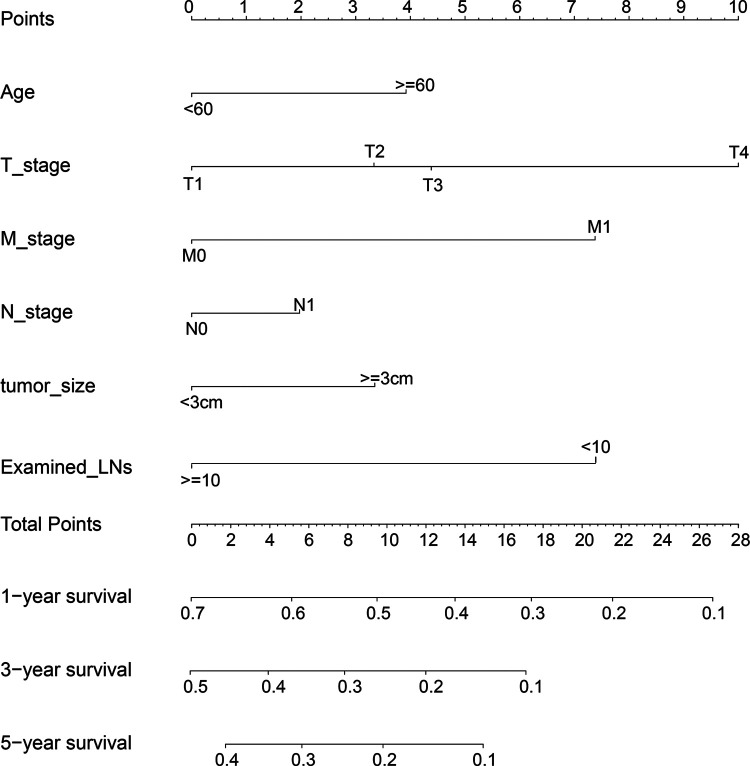
Model predicted 1-, 3- and 5-year CSS based on the number of ELNs.

To determine the most suitable features to build a nomogram, we performed a multivariate cox analysis, and the independent prognostic factors included age, tumor size, TNM stage and ELN (Figure 4). Patients who were aged ≥60, with tumor size >3 cm, or with lymph node metastasis had a worse prognosis, while patients with ELNs >10 have a better prognosis. After multivariate cox analysis, compared to the white race, the black race was a risk factor for survival, however, the other races were not associated with survival. Therefore, the race was excluded. The record of marital status contained much uncertain information, hence it is hard to accurately identify marital status as an independent factor. Then a nomogram predicting prognosis was constructed based on the results above (Figure 5). As shown in the survival model, T stage had the greatest impact on prognosis, followed by ELNs, distant metastasis, tumor size, and age, while LNM did the least effect on prognosis.

### Nomogram validation

**Figure 6:**
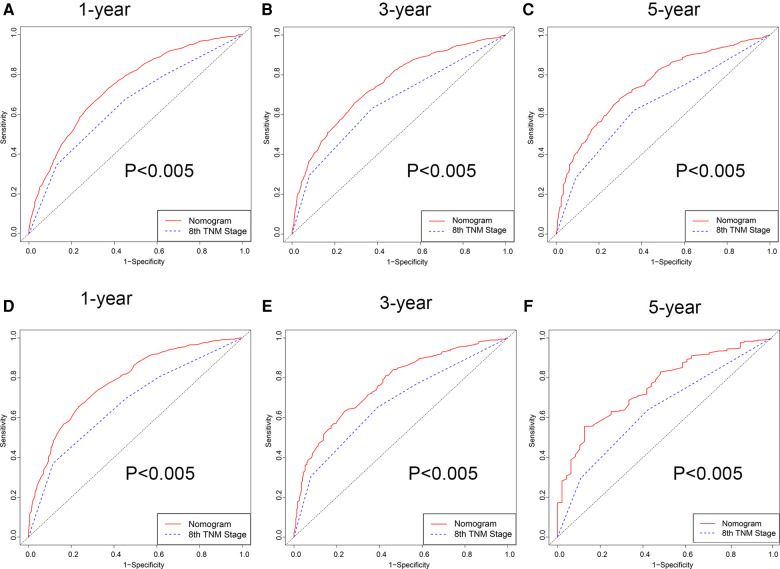
ROC curve of the nomogram and 8th TNM stage. (**A–C**) 1-, 3- and 5-year in the 2004–2009 cohort. (**D–F**) 1-, 3- and 5-year in the 2010–2015 cohort.

**Figure 7:**
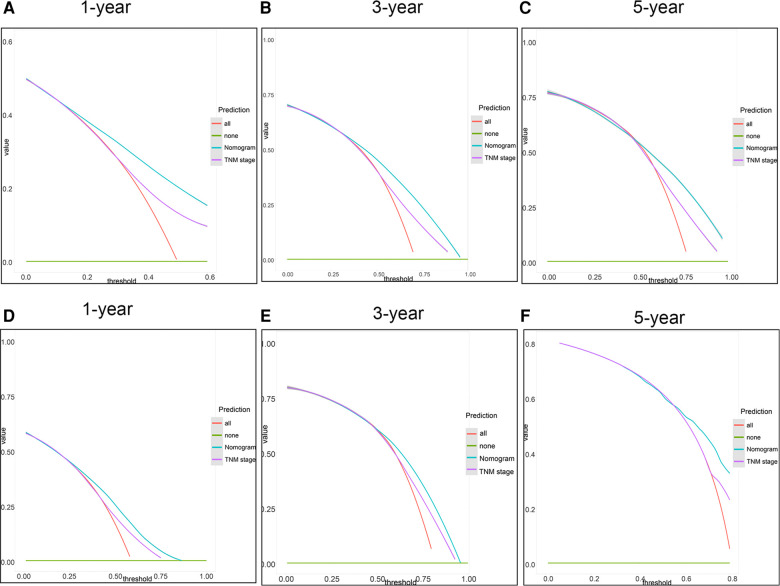
DCA of the nomogram and the 8th TNM stage. (**A–C**) 1-, 3- and 5-year points in the 2004–2009 cohort. (**D–F**) 1-, 3- and 5-year in the 2010–2015 cohort.

**Figure 8:**
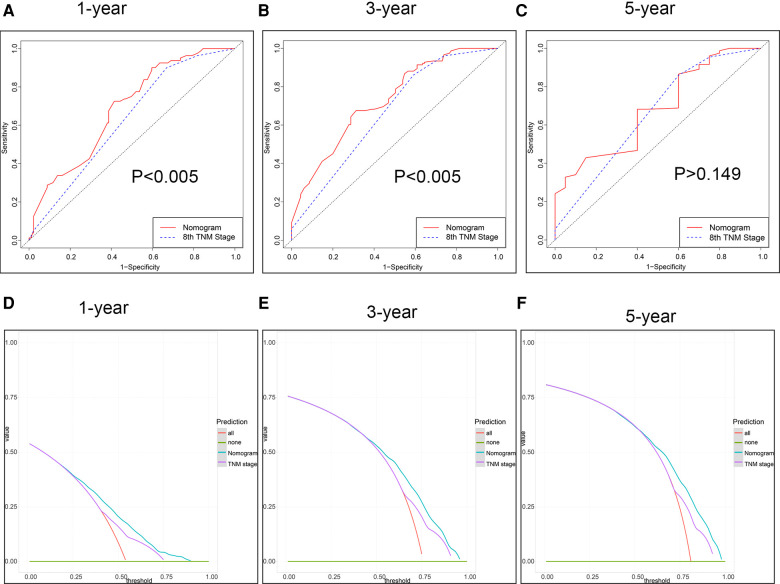
The ROC curve and DCA of the model from our centre. (**A–C**) ROC curve for 1-, 3- and 5-year. (**D–F**) DCA for 1-, 3- and 5-year survival.

Firstly, in our training cohort, the C-index of the nomogram model has a value of 0.708 which ranged from 0.678 to 0.753, which were better than that of the 8th TNM staging system (Table 3). The external validation cohort also showed our model had a good C-index value (0.687, ranging from 0.601 to 0.734). In line with the training cohort and external validation, the result of analyzing data from our center also demonstrated nomogram model with a C-index value of 0.652 was better than that of the traditional 8th TNM stage of which the C-index was 0.604 (Table 3). For specificity and sensitivity of diagnosis, the model also outperformed TNM stage in both cohorts (*P* < 0.001, Table 3 and Figures 6A–C) and external cohort (*P* < 0.001, Table 3 and Figures 6D–F). Finally, we performed DCA to compare the clinical usability, finding nomogram showed a greater benefit compared to the TNM staging system for predicting the CSS with different survival time (Figure 7). Furthermore, the above results were additionally testified by data from our center. As shown in Table 3 and Figures 8A–C, the nomogram model was better than the TNM stage for predicting survival (*P* < 0.05). However, the difference in predicting 5-year survival had no significance (*P* = 0.149). The results of DCA also showed nomogram was more favorable for clinical decision and assessment (Figures 8D–F).

**Table 3:** Prediction accuracy of modified model vs. TNM stage in survival of ESCC patients.

Variable	Value (95%CI)		
	Internal validation	External validation	Validation in our center
C index for nomogram	0.708 (0.678–0.753)	0.687 (0.601–0.734)	0.652 (0.589–0.703)
C index for TNM stage	0.601 (0.573–0.656)	0.605 (0.563–0.659)	0.604 (0.561–0.673)
1-year AUC for nomogram	0.753 (0.711–0.821)	0.761 (0.715–0.831)	0.686 (0.621–0.752)
3-year AUC for nomogram	0.761 (0.712–0.813)	0.753 (0.659–0.818)	0.73 (0.67–0.788)
5-year AUC for nomogram	0.783 (0.753–0.848)	0.75 (0.753–0.847)	0.679 (0.548–0.798)
1-year AUC for TNM stage	0.653 (0.611–0.701)	0.641 (0.605–0.715)	0.625 (0.574–0.675)
3-year AUC for TNM stage	0.701 (0.675–0.784)	0.687 (0.655–0.738)	0.662 (0.609–0.715)
5-year AUC for TNM stage	0.733 (0.613–0.781)	0.685 (0.643–0.727)	0.655 (0.545–0.765)

## Discussion

ESCC is the predominant histologic subtype of EC over the world, while adenocarcinoma is mainly distributed in North America and Europe. ESCC was derived from an epithelial cell of the mucosa, which was often stimulated due to alcohol and smoke (5). Radical surgery is considered the preferable therapeutic method, especially for some minimally invasive surgery such as endoscopic surgery (25). However, the long-term survival was still low because of high recurrence or distant metastasis. Therefore, radical resection and adequate lymph node dissection were critical. This study shows that the number of ELNs has a significant impact on the prognosis of ESCC patients. Moreover, we determined the optimal demarcation of ELNs was 10 using X-tile software and divided patients into two groups: ≤10 ELNs and >10 ELNs. At the same time, we performed multivariate regression analysis and built a nomogram model, of which the process was credible and accurate (26). Furthermore, the nomogram was validated by the training cohort and two external cohorts, suggesting it was superior to the traditional 8th TNM staging system as far as clinical usefulness was concerned.

It is well-known that ELNs are one of the important factorsassociated with patients’ prognosis, which was also demonstrated by many previous studies (27, 28). Several studies found the number of ELNs (>15) affects the prognosis of ESCC patient (29). In our study, we found that the best cut-off value of ELNs was 10, which was in line with other studies (15, 30, 31). Considering the AUC value of ROC, the nomogram performed well with a value of 0.7 and was better compared to the 8th TNM staging (32, 33). In addition, some researchers put forward other different views on TNM stage (15, 34, 35). By figuring out the C-index value and performing tdROC and DCA, we demonstrated nomogram was more effective on clinical usability compared to the TNM staging system, which was also tested by many previous studies (32, 33).

In our model, we totally included age, TNM stage, tumor size, and ELNs to build the model. Usually, the pathological grade was considered as an independent factor for patients’ survival. However, we excluded it according to multivariate analysis (36). We thought the main reason was the limited sample of different pathological subtypes. Regarding the cut-off value in our study, of course, different studies reported diversely. As for stage IV of ESCC, a study thought 18 ELNs were necessary for determining accurate staging and improving survival (37), while another study indicated that 15 ELNs at least were favorable r for patients’ survival (38). However, removing lymph nodes and assessing LNM depended on the surgeon and pathological clinicians to some extent (14). Therefore, the differences in studies may be due to the heterogeneity of the study population. Although there were similar studies focused on the cut-off value of ELN (29), our study further constructed a predicting model of survival based on the number of ELN, which made the study more clinically meaningful. To some extent, we could assess the survival of patients after surgery according to the nomogram.

Our study also has some limitations that cannot be ignored. First, we excluded patients with missing data such as the TNM staging and pathological grade, leading to the increased selection bias. Next, our manuscript has not included other characteristics both in the SEER database and in our own data, such as hematological biomarkers and molecular parameters, which made our model limited. Next, in fact, we found the SEER data showed of 85% patients with less than 10 ELN, which was inconsistent with our data, affecting our analysis of survival in general. But we checked other studies about SEER data, we found there was a similar rate of less than 10 ELNs (9). Moreover, the low rate of ELN would underestimate the stage of the tumor, decreasing the reliability of our study. Finally, whether patients from the SEER database received chemotherapy after surgery or radiotherapy was not known to us, which did make a great difference for our analysis. However, in our data, we included the information about chemotherapy, making an explanation to problems to some extent. Also, as for the result of own data, we found the nomogram model was similar to the TNM stage for predicting 5-year survival (*P* = 0.149). We thought the limited samples of patients with 5-year survival were the main reason because the nomogram model performed well in the internal and external validation group which had sufficient patients with 5-year survival. Of course, this hypothesis needs to be proven by enrolling a larger sample of patients in the future.

## Conclusions

In addition to ELNs was an independent protective factor, variables including age, tumor size, and TNM stage were the independent risk factor for CSS according to the results of multiple statistical analyses. The number of ELNs was more favorable when it was more than 10. More than 10 ELNs are helpful to evaluate the survival of ESCC patients. Based on this, an improved model for predicting the prognosis of ESCC patients was constructed and could serve as an assistive tool for survival evaluation compared to the 8th TNM staging system.

## Data Availability

The raw data supporting the conclusions of this article will be made available by the authors, without undue reservation.
