# Supplementary figures and images for: A modified survival model for patients with esophageal squamous cell carcinoma based on lymph nodes: A study based on SEER database and external validation
Source: Front Surg. 2022 Sep 7;9:989408. doi: 10.3389/fsurg.2022.989408 (PMC9489949; doi:10.3389/fsurg.2022.989408)

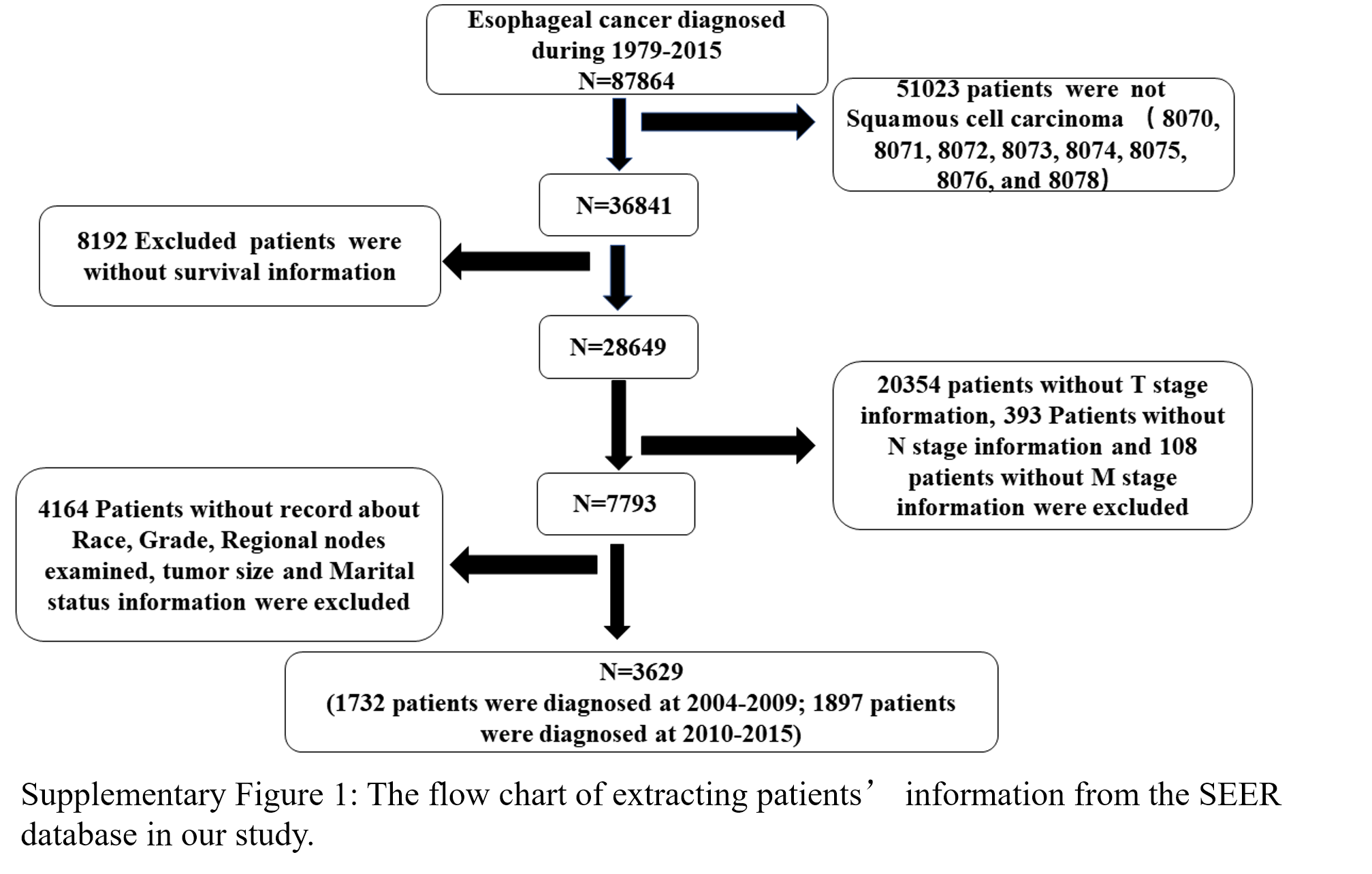

Supplement: Supplementary file 1 [file Image_1_v1.tif]

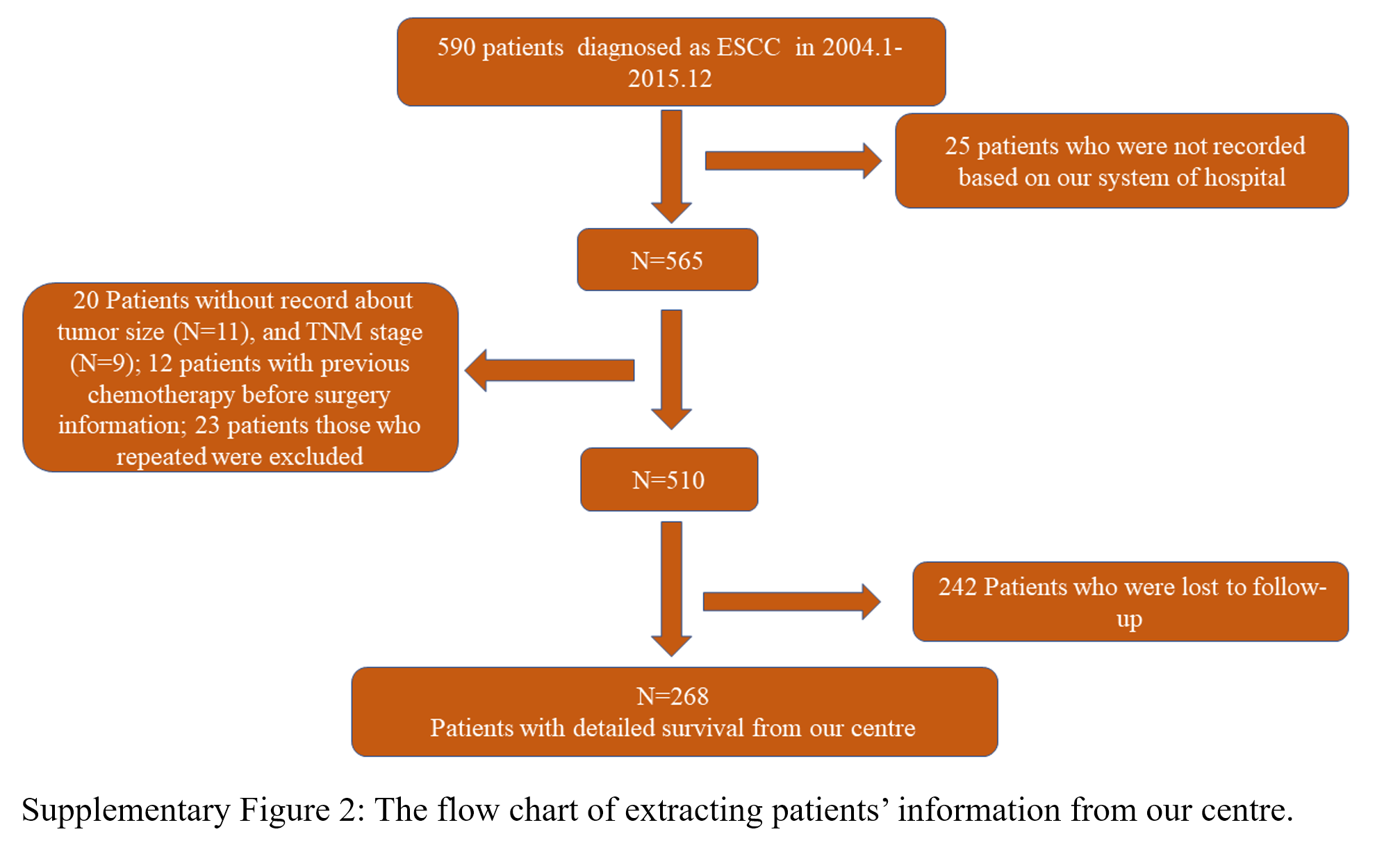

Supplement: Supplementary file 2 [file Image_2_v1.tif]

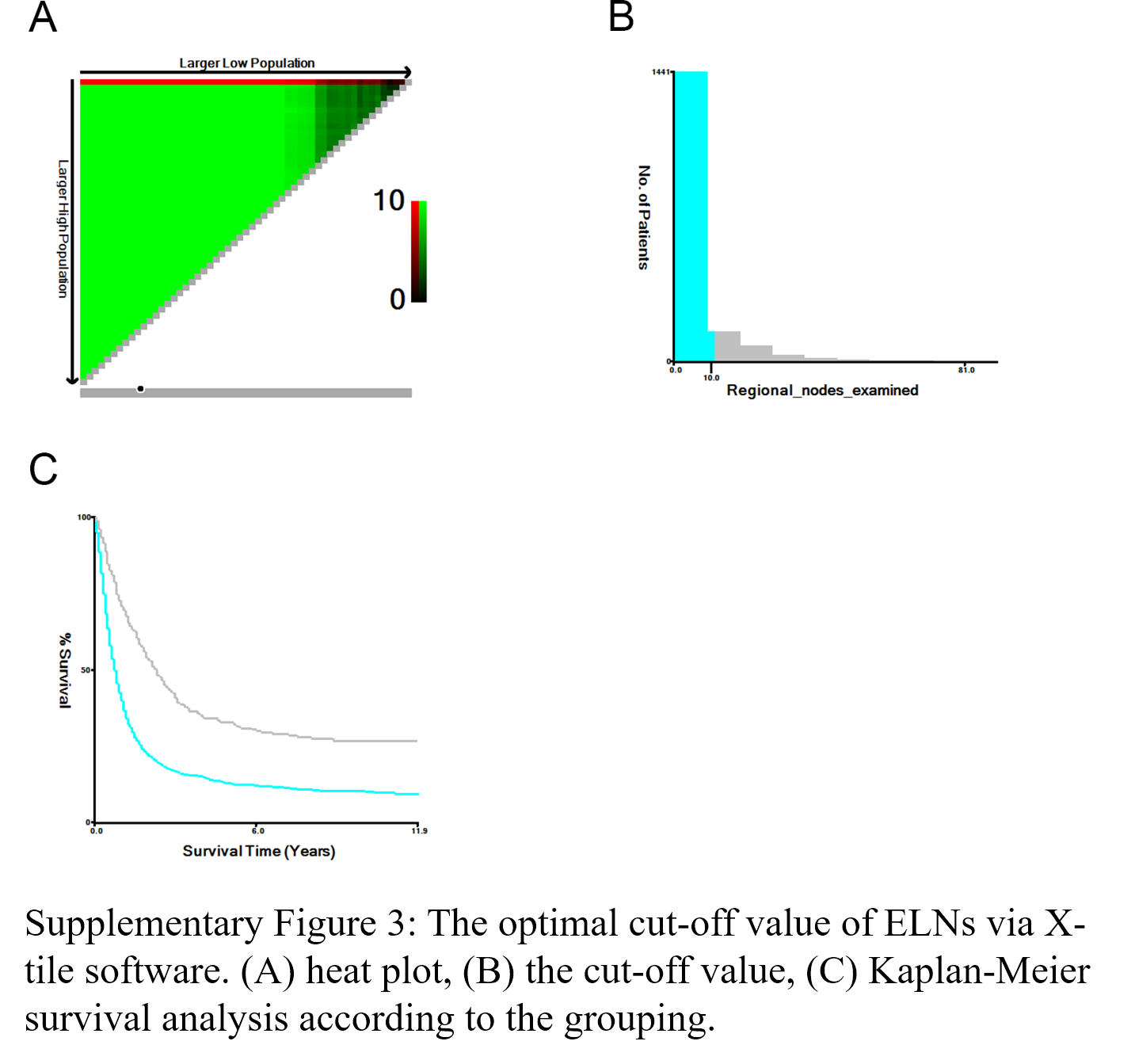

Supplement: Supplementary file 3 [file Image_3_v1.tif]
